# 3D Printing and Virtual Surgical Planning in Craniofacial and Thoracic Surgery: Applications to Personalised Medicine

**DOI:** 10.3390/jpm15090397

**Published:** 2025-08-25

**Authors:** Freddy Patricio Moncayo-Matute, Jhonatan Heriberto Vázquez-Albornoz, Efrén Vázquez-Silva, Ana Julia Hidalgo-Bravo, Paúl Bolívar Torres-Jara, Diana Patricia Moya-Loaiza

**Affiliations:** 1 Grupo de Investigación en Nuevos Materiales y Procesos de Transformación (GIMAT), Department of Mechanical Engineering, Universidad Politécnica Salesiana, Sede Cuenca EC010102, Ecuador; fmoncayo@ups.edu.ec (F.P.M.-M.); ptorresj@ups.edu.ec (P.B.T.-J.); dmoyal@ups.edu.ec (D.P.M.-L.); 2 Plastic and Reconstructive Surgery Department, Hospital del Río, Cuenca EC010109, Ecuador; jhvaplastic@gmail.com (J.H.V.-A.); anajuliahidalgob@gmail.com (A.J.H.-B.)

**Keywords:** additive manufacturing, cutting guide, surgical planning, medical image, reverse engineering

## Abstract

**Background/Objectives:** The application of additive manufacturing in medicine, and specifically in personalised medicine, has achieved notable development. This article aims to present the results and benefits of applying a comprehensive methodology to simulate, plan, and manufacture customised three-dimensional medical prosthetic devices for use in surgery to restore bone structures with congenital and acquired malformations. **Methods:** To digitally reconstruct a bone structure in three dimensions from a medical image, a segmentation process is developed to correlate the anatomical model. Then, this model is filtered using a post-processing step to generate stereolithography (STL) files, which are rendered using specialised software. The segmentation of tomographic images is achieved by the specific intensity selection, facilitating the analysis of compact and soft tissues within the anatomical region of interest. With the help of a thresholding algorithm, a three-dimensional digital model of the anatomical structure is obtained, ready for printing the required structure. **Results:** The described cases demonstrate that the use of anatomical test models, cutting guides, and customised prostheses reduces surgical time and hospital stay, and achieves better aesthetic and functional results. Using materials such as polylactic acid (PLA) for presurgical models, appropriate resins for cutting guides, and biocompatible materials such as polyether ether ketone (PEEK) or polymethylmethacrylate (PMMA) for prostheses, the described improvements are achieved. **Conclusions:** The achievements attained demonstrate the feasibility of applying these techniques, their advantages and their accessibility in Ecuador. They also reinforce the ideas of personalised medicine in the search for medical treatments and procedures tailored to the needs of each patient.

## 1. Introduction

Additive manufacturing (AM) is applied in many areas of social development. Among these, public health stands out. This technology is significantly impacting the medical sector, helping to produce customised and complex medical devices, improving the results of surgical procedures on patients and optimising their precision.

Additive manufacturing facilitates the production of customised implants, i.e., implants tailored to the individual anatomy of the patient receiving the implant. Such customisation measures have been demonstrated to ensure an adequate fit level and improve surgical outcomes [1,2,3]. Furthermore, technological advances in this field demonstrate great results in enhanced patient comfort and satisfaction [4,5]. Additionally, the fabrication of intricate structures and complex geometries, which are challenging to replicate through conventional manufacturing methodologies, is attainable [3,6].

A recent literature review indicates a substantial increase in scientific publications concerning surgical applications of AM and 3D printing. Cranio-maxillofacial surgery accounted for the second highest percentage of these publications. As additive manufacturing technology improves and becomes more affordable, its use in plastic and reconstructive surgery is also expanding (see references [7,8]).

In such applications, the materials employed encompass biocompatible metals, such as titanium alloys, polymers and bio-composites, which possess the capacity to emulate (and even surpass) the mechanical properties of bone [9,10,11]. A variety of additive manufacturing techniques are under development to produce medical devices for a range of specific applications. The following processes are included: direct laser sintering of metals, stereolithography, selective laser sintering, modelling and fused deposition manufacturing or fused deposition modelling (FDM) [11,12,13,14,15,16,17,18].

Image integration and reverse engineering processes are of pivotal significance in the medical device manufacturing domain. Additive manufacturing has been demonstrated to successfully integrate with medical imaging techniques (e.g., computed tomography (CT) scans) to create accurate three-dimensional digital models of the patient’s specific anatomy. These models are integrated into design methodologies and the additive manufacturing processes themselves, intending to generate surgical instruments digitally, anatomical test models to simulate procedures, implants and surgical guides [3,6]. Reverse engineering is defined as the process of creating a three-dimensional digital model of an object or organ of a patient’s anatomy from medical imaging data [6]. These models are indispensable for designing perfectly fitting implants, reducing the risk of implant failure and improving post-intervention outcomes. The extent of customisation ensures that devices possess optimal dimensions, form, and mechanical properties, which are particularly advantageous in complex surgeries, such as craniomaxillofacial and orthopaedic surgeries [1,19,20,21,22].

Medical images (e.g., magnetic resonance imaging (MRI), CT scan, X-ray, ultrasound) can play a key role in fabricating customised medical devices and implants through additive manufacturing technology. This process comprises a series of stages in which image data play an important role. In the context of custom implants, for instance, CT and MRI are the techniques most frequently employed to obtain detailed images of the patient’s anatomy. These images are then converted into digital models that accurately represent the patient’s unique anatomical features [23,24,25]. The subsequent processing of the acquired image data is facilitated by specialised software (e.g., OsiriX Imaging Software (https://www.osirix-viewer.com/), 3D Slicer 3.5, Mimics 21.0, Magics 2.0, 3D Doctor, InVesalius 3.0, among others), which enables the creation of a 3D digital model. This model is then exported in a standard triangulated language (or stereolithography) (STL) format for utilisation in AM technologies [1,15,19,23,24]. The implant parameter verification, including shape, external dimensions and internal structure (e.g., porosity), is also performed by imaging techniques such as CT [16,17]. In the context of manufacturing, the thickness of the layer during production is a critical factor, as it can influence the mechanical properties and the precision of the implant [26].

The necessity for enhanced visualisation of surgical processes and outcomes has precipitated the emergence of anatomical test models, patient-specific surgical guides (in addition to customised 3D printed implants) [8]. These processes involve several critical considerations to ensure accuracy, safety and efficacy. Cutting guides must be adapted to the patient’s anatomy to ensure accurate fit and effective surgical outcomes [2,27,28]. The materials utilised must demonstrate an adequate degree of biocompatibility to circumvent the occurrence of adverse reactions. An anatomical test model used for simulating surgery does not necessitate the utilisation of a biocompatible material in its fabrication. In the case of an implant intended to be permanent, it is essential to utilise a material that exhibits the highest degree of biocompatibility. The most common materials used in this process are polymers and metals, such as titanium and polyether–ether–ketone (PEEK) [13,27]. The clear procedural establishment, standards design and guidelines are also of great importance. This includes ensuring that the cutting guide can be uniquely positioned and stably fixed during surgery [27,29].

Before clinical implementation, the ergonomic effectiveness and ease of use of the device must be evaluated in simulated environments. This is an integral component of surgical planning, enabling surgeons to meticulously try the procedure, anticipate potential challenges and refine the procedural approach. It is evident that adopting a comprehensive approach results in enhanced precision and efficiency during surgical procedures. This approach enables the selection and optimisation of materials based on their biocompatibility, strength and suitability for specific applications [19,21,22,30,31,32].

Integration of these processes, namely reverse engineering with AM and the study of materials and imaging techniques, facilitates the design of implants with complex geometries and reticular structures that promote osseointegration and reduce device stiffness. This facilitates enhanced biomechanical performance and longevity (see references [1,33]).

The utilisation of custom implants and cutting guides, which have been reverse-engineered and designed using AM, has demonstrated results in superior fit and enhanced performance compared to conventional implants. This has been demonstrated to result in improved patient outcomes. Surgical planning and simulation have been shown to reduce the risks associated with complex surgeries. As evidenced by the existing bibliography, the expense of the initial set-up can be high. However, the overall cost is reduced through fewer complications and shorter surgery times [1,3,19,21,22,31]. Nevertheless, considerable regulatory requirements are in place for the approval of medical devices produced by AM, which has the potential to impede the process of adoption [4,13].

This article describes seven cases in which the entire process of obtaining anatomical test models for surgical simulation, designing surgical cutting guides, and custom-made cranial and clavicular implants was carried out, all manufactured using additive printing techniques. The objective of this analysis is to clarify any potential ambiguities or unresolved questions that may remain concerning these technologies. What are the advantages that can be attributed to them? It is imperative to ascertain whether the advantages of this procedure are sufficient to compensate for any potential disadvantages that may persist.

However, the questions that may still be unanswered do not minimise the achievements in the field that closely connects mechanical engineering, materials science, and medicine. The cases presented are examples of the precision medicine application. The anatomical models for surgical planning, cutting guides, and implants themselves are custom-made according to the medical requirements of each patient who benefits from each type of device. The information used to apply the comprehensive methodology developed by the research team for additive manufacturing and subsequent use of the different devices based on anatomical dimensions is entirely individual. This contributes to optimising efficacy and minimising side effects.

The article structure is as follows. Section 2 provides a detailed description of the materials employed and the applied methodology in the surgical interventions that are documented herein. Section 3 presents a general description of each case, including the diagnosis, the procurement of the anatomical test model, the surgical planning, the approach to the surgical process, and the postoperative follow-up. The results are then presented and discussed in general terms. Section 5 of this study offers some conclusions.

## 2. Materials and Methods

To digitally reconstruct a bone structure in three dimensions from a medical image, a segmentation process is carried out to correlate the anatomical model. This model is filtered using a post-process to generate stereolithography (STL) files exported from 3D Slicer 3.2 software https://www.slicer.org (accessed on 14 July 2025), which are rendered using Autodesk Meshmixer 3.5 software https://www.meshmixer.com (accessed on 14 July 2025). The segmentation of tomographic images is achieved by the specific intensity selection, known as Hounsfield Unit (HU). This intensity measures the attenuation coefficient in the grey scale, facilitating the analysis of compact and soft tissues within the designated anatomical region [34]. A thresholding algorithm is applied to obtain a three-dimensional digital model of the anatomical area of interest, ready for printing. In the anatomical test model fabrication for surgical planning, the additive manufacturing technology FDM is employed, utilising a Creality CR-X Pro 3D FDM printer (Shenzhen Creality 3D Technology Co., Ltd. (Shenzhen, China)).

The material employed for the manufacturing of anatomical test models is polylactic acid (PLA), fabricated by the Creality brand. The long-lasting implants, where required, are manufactured with polymethylmethacrylate (PMMA) applying the mould-forming technique.

The route code reading, processing and generation starts from the STL file of the segmentation. The fabrication of the printed object is facilitated by Creality Slicer, version 1.2.3, an open-source and cost-free software.

Table 1 describes the characteristics of the FDM technology. The values of the manufacturing parameters are adjusted to obtain the best performance in the process.

The printing parameters are set based on the geometric characteristics, size, surface quality, the anatomical model functionality, and the type of material used. The mechanical characteristics of the employed polymers are outlined in Table 2. It should be noted that the PLA is not biocompatible and is employed exclusively for the anatomical test models.

It is essential to note that, within the context of the care process for each patient, the phases of medical device design and simulated surgery must be integrated. The design is supported by tomographic image analysis, which provides anatomical test models, patient-specific cutting guides, and custom implants (if necessary). In summary, the segmentation algorithms have been shown to facilitate the identification of the bone structure in need of restoration. Furthermore, the performance of measurements to manufacture cutting guides has been demonstrated to be possible for a range of surgical procedures, including tumour excision with negative margins, following a previous tomographic assessment.

Concerning the regulations for this procedure, it should be noted that all of the cases presented here were part of a research project. In such a condition, Ecuadorian national legislation stipulates that informed consent is required from the patient (or the patient’s parents if the patient is a minor) (Resolución ARCSA-DE-2023-033-AKRG Reforma a la Normativa Técnica Sanitaria para el Registro Sanitario de Dispositivos Médicos de Uso Humano y de los Establecimientos en donde se fabrican: ARCSA Regulations—Instructions https://www.controlsanitario.gob.ec/documentos-vigentes/ (accessed on 21 July 2025)). Therefore, signed documents of acceptance for participation in the project are available for all treated patients.

### 2.1. Approach

The research adopts a clinically applicable approach, supported by a descriptive observational methodology, with integration of virtual surgical planning and 3D printing technologies. From tomographic images, personalised anatomical models and specific surgical guides are developed for each case, evaluating the surgical precision, safety of the procedure and comprehensive clinical results.

### 2.2. Process

A series of seven consecutive clinical cases is presented, selected from patients with complex craniofacial and thoracic pathologies. No patients were excluded if they met the minimum clinical and imaging criteria required for accurate anatomical segmentation and custom fabrication. All procedures were performed within the framework of an interdisciplinary program of clinical application of 3D technologies.

The process included obtaining tomographic images, segmentation with the help of free-access software, digital anatomical modelling, surgical guides design, personalised implants, and subsequent manufacturing using 3D printing in materials such as PLA (for models), Resin (for the guides) and PMMA and PEEK (for definitive implants). Validation was developed jointly between the medical team and engineers.

The interventions were planned using anatomical test models, printed in 3D, and executed with the support of personalised surgical guides. The surgeries had an average duration of between 1 and 2 h, with controlled bleeding, within the expected physiological ranges according to age and type of procedure. No significant intra- or postoperative complications were reported.

The cases described in the following section were not selected following a defined criterion. They only constitute representative cases of the possibilities that additive manufacturing opens up within personalised medicine.

In the treatment of these patients, a methodology was applied in collaboration through a research project on additive manufacturing and its applications in personalised surgical devices between academia and the public health sector, specifically between the Research Group on New Materials and Transformation Processes (GIMAT, acronym in Spanish) of the Faculty of Mechanics in the Universidad Politécnica Salesiana and a medical team from the Hospital del Río, made up of a neurosurgeon specialist and two specialists in maxillofacial and aesthetic surgery. Both institutions are from Cuenca City in Ecuador.

In this context, the needs of each patient to support each surgical treatment were provided by the hospital institution in terms of personalised medical devices to be designed and manufactured. This is the reason why there is no control group.

## 3. Virtual Surgical Planning, Computer-Aided Design and Manufacturing Technologies in Clinical Cases

This section presents the results obtained for seven cases, each with a distinct pathology and sequelae, necessitating intervention related to the developed methodology.

### 3.1. Case 1: Skull Trauma Sequel

#### 3.1.1. Diagnosis and Analysis

**Figure 1:**
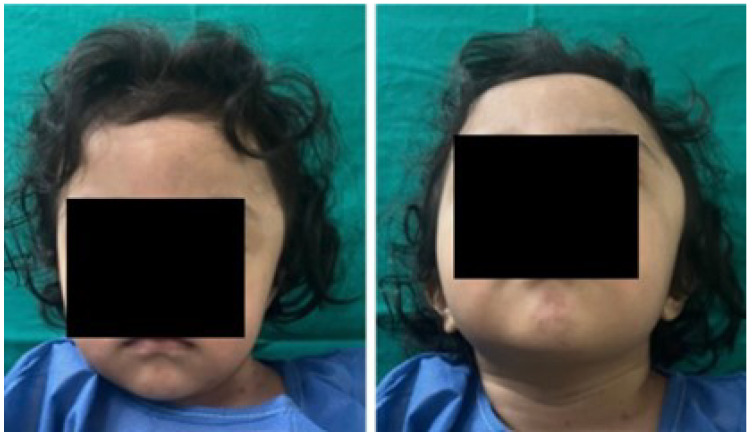
Left temporoparietal prominence and ocular proptosis.

**Figure 2:**
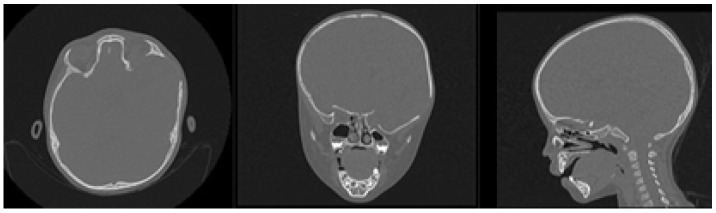
Computed tomography (CAT). Left temporoparietal cranial defect, solution of continuity in the roof of the orbit and temporoparietal region.

A 2-year-old female patient attended the consultation due to prominence in the temporal region as a result of cranioencephalic trauma. Physical examination revealed the prominence of the left temporal bone and ocular proptosis on the same side (see Figure 1). A computerised axial tomography (CAT) with three-dimensional reconstruction revealed a solution of bone continuity in the orbital roof and region of the left temporal squama, with front-orbital and temporal encephalocele (see Figure 2).

#### 3.1.2. Surgical Planning

**Figure 3:**
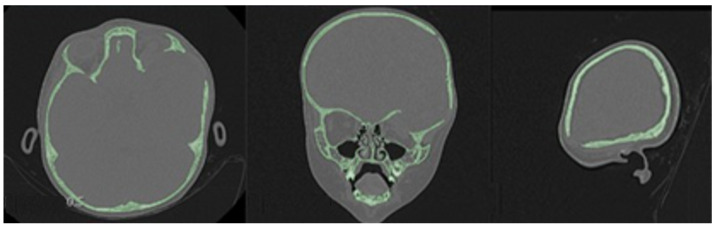
Selection of the HU intensity (corresponding to the necessary bone density) from the tomographic image.

**Figure 4:**
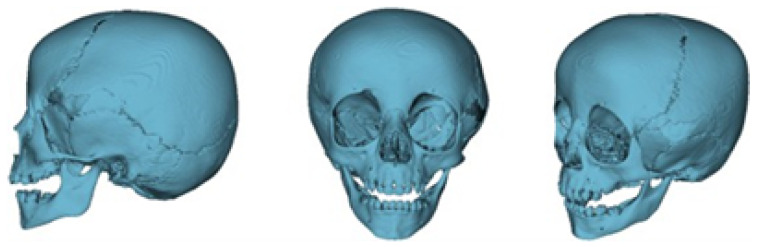
Bone segmentation of the anatomical model with the cranial defect.

The defects identified were corroborated by both the patient’s medical history and physical examinations, as well as by tomography. The tomographic images were then segmented to reconstruct the left temporo-parietal cranial bone tissue. The density of compact cranial tissue was established using a range of 180 to 2000 HU (see Figure 3 and Figure 4).

#### 3.1.3. Planning and Printing Anatomical Model

**Figure 5:**
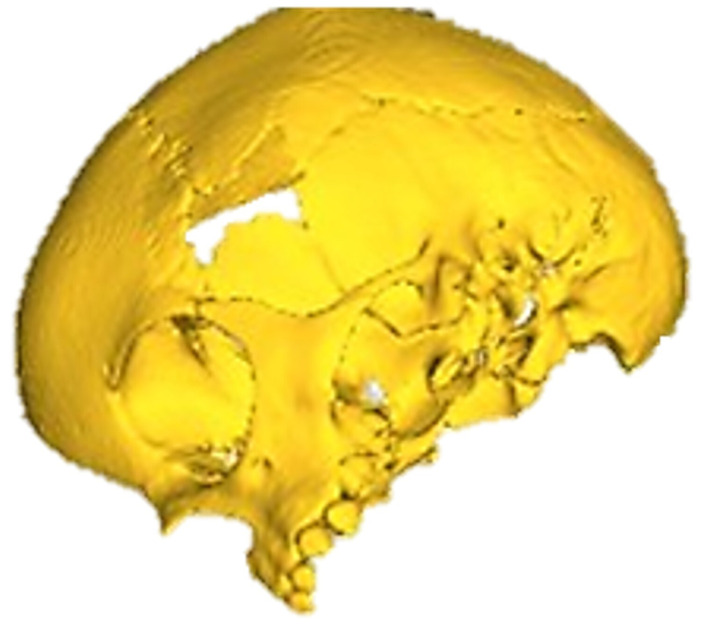
Preparation of the 3D-printing model.

**Figure 6:**
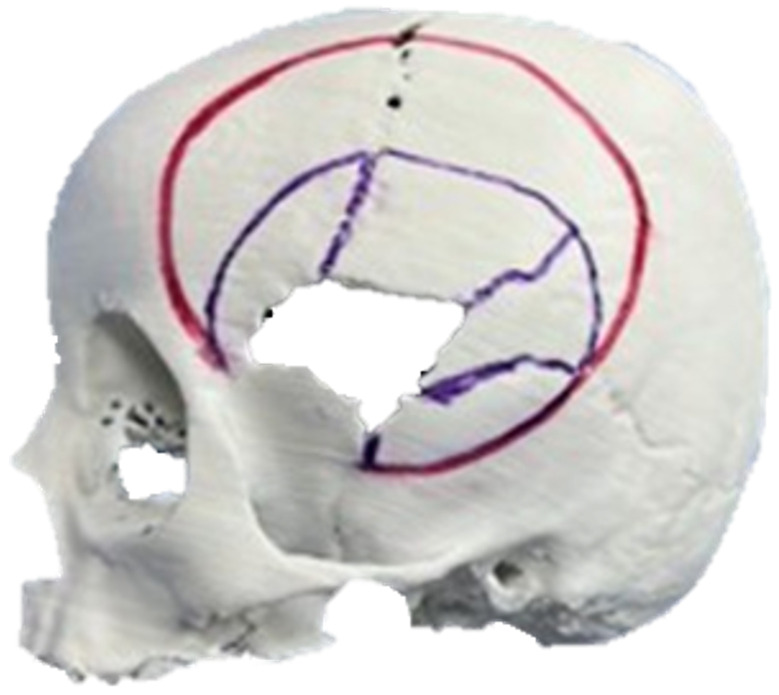
Three-dimensional anatomical model of the skull and cutting plan.

The segmentation process facilitated the correlation of the digital anatomical model (in which the left temporoparietal defect was identified) and the physical anatomical model for simulated surgical planning (see Figure 5 and Figure 6).

#### 3.1.4. Intraoperative Approach

**Figure 7:**
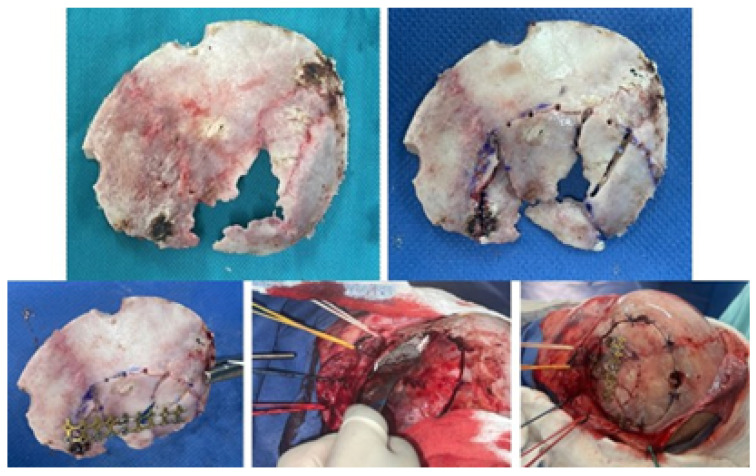
Resected bone complex (**top left**). Bone complex with osteotomies (**top right**). Union of osteotomies and reconstruction of the temporoparietal bone defect with osteosynthesis (**bottom left**). Placement of orbital roof prosthesis for its reconstruction (**bottom centre**). Placement of reconstructed bone complex (**bottom right**).

Following a rigorous testing and planning process in the 3D model, the actual intervention was carried out with the previously chosen technique. During the surgical procedure (Figure 7), cranial osteotomies were performed, in addition to in-bloc resection of the entire affected bone complex (Figure 6, red line). This complex consists of the orbital roof, zygomatic-temporal region, and temporoparietal region. Subsequently, reconstructive osteotomies and the placement of prostheses or osteosynthesis material were performed, as required (purple lines in Figure 6).

#### 3.1.5. Postoperative

**Figure 8:**
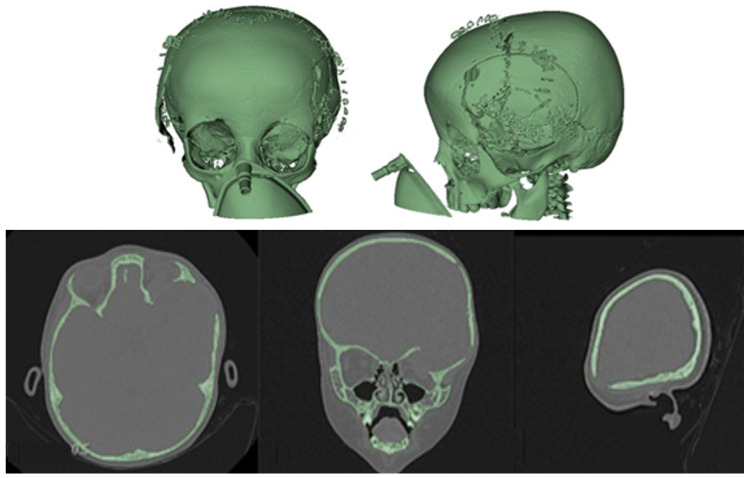
Immediate postoperative tomographic control. The correction of the cranial defect, temporoparietal encephalocele and the roof of the orbit are observed.

**Figure 9:**
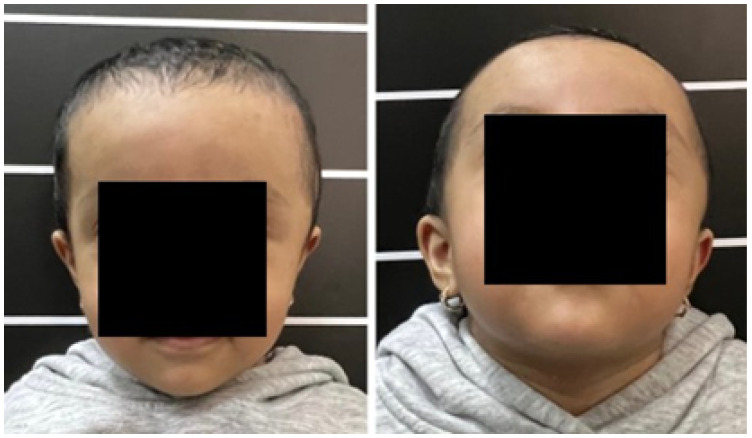
Significant reduction in ocular proptosis and temporoparietal prominence is observed.

The surgical outcomes were corroborated through a combination of tomographic analysis, digital photography, and physical examination (see Figure 8 and Figure 9). These post-surgical assessments revealed a substantial enhancement in the temporoparietal bone, a finding that was instrumental in guiding the surgical planning process.

### 3.2. Case 2: Skull Trauma Sequel

#### 3.2.1. Diagnosis and Analysis

**Figure 10:**
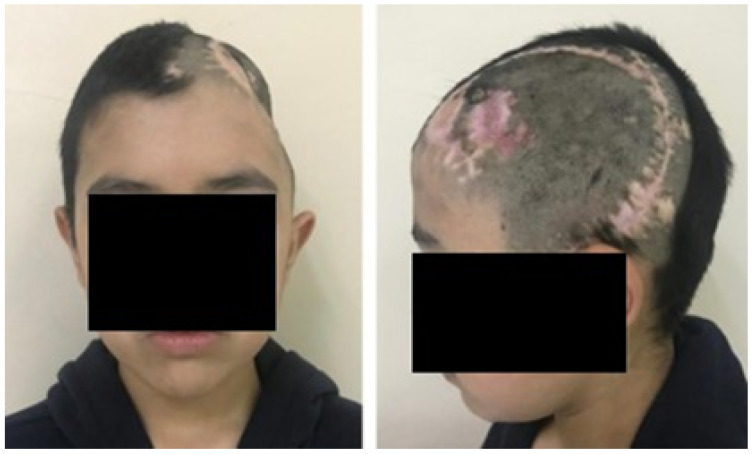
Collapse in the left temporo-parieto-occipital region.

**Figure 11:**
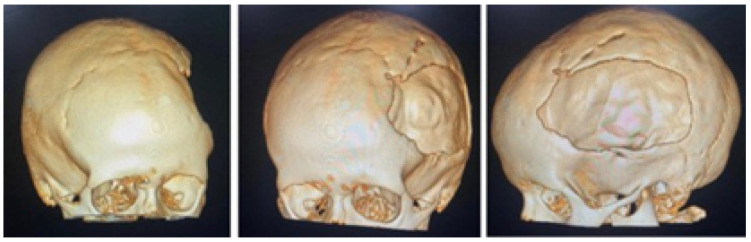
TAC: sinking in the temporo-parieto-occipital region (**left**). Solution of bone continuity in the affected area (**centre**-**right**).

The patient was a 10-year-old male who sustained a cranial collapse in the left temporal-parietal-occipital region. This is a sequelae of cranioencephalic trauma, with multiple surgical interventions having been performed. A physical examination was conducted, which revealed subsidence in the temporo-parieto-occipital region, accompanied by some scars. Cerebral activity manifests itself externally, as evidenced by the visible transmission of light from the brain to the skin (see Figure 10). Upon tactile examination, the irregular edges of a bone defect resulting from an anterior craniotomy were palpable. A CT scan with 3D reconstruction was performed, where the solution of bone continuity in the affected area can be seen (Figure 11).

#### 3.2.2. Surgical Planning

**Figure 12:**
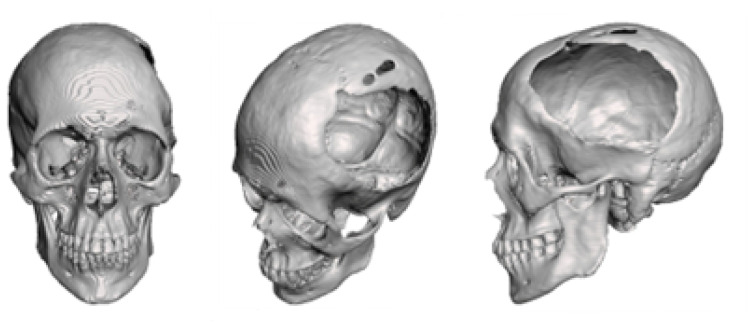
Bone segmentation of the anatomical model from the tomographic image.

Reconstruction of the defects found, both in the anamnesis and in the physical and radiological examinations, was performed applying 3D digital restoration (Figure 12) and manufacturing of the anatomical test models with the help of stereolithography.

#### 3.2.3. Anatomical Model Printing

**Figure 13:**
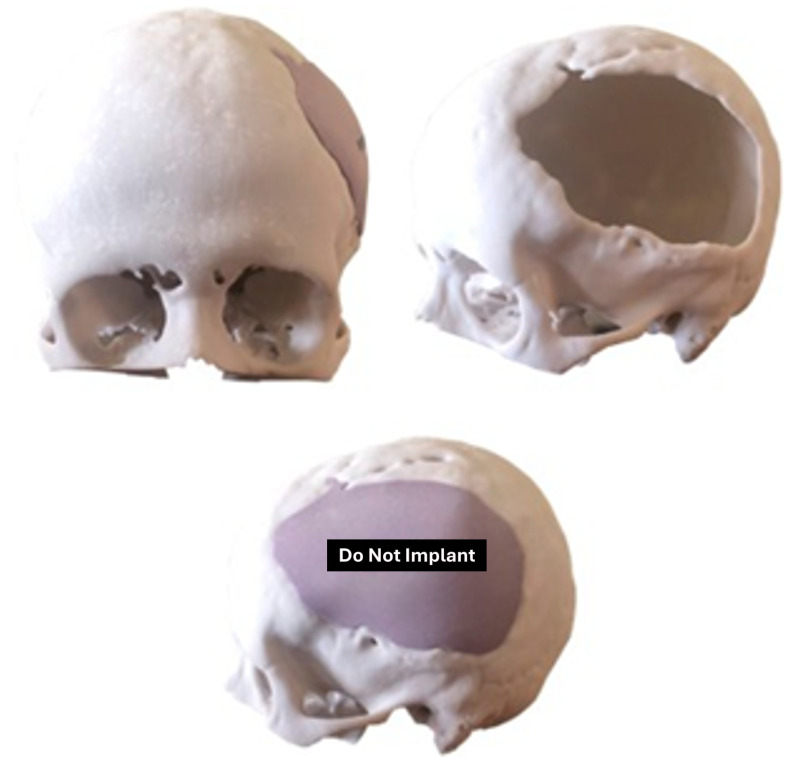
Three-dimensional anatomical model of the skull and non-implantable prosthesis (only for planning).

The 1:1 scale digital model facilitates the production of a precise and customised prosthesis for the particular bone defect, obviating the necessity of a bone tissue donor area creation. As illustrated in Figure 13, the cranial anatomical model and the customised implant prototype were positioned accordingly. The methodology described in [35] was followed for the prosthesis design and manufacture.

#### 3.2.4. Intraoperative Approach

**Figure 14:**
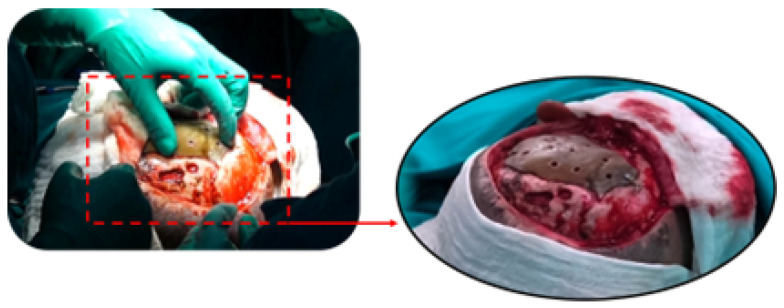
Three-dimensional patient-specific implant placement (**left**). Detail of implant fixation (**right**).

The surgery was simulated, and in this trial, the prosthesis was tested in the 3D model. The surgical procedure was then conducted in a living subject, employing the selected approach determined through simulation. The final prosthesis was placed and manufactured using medical-grade PMMA. This approach enabled the reconstruction of the bone defect and the recovery of the patient’s cranial aesthetics. As illustrated in Figure 14, the surgical process was observed at various stages.

#### 3.2.5. Postoperative Results

**Figure 15:**
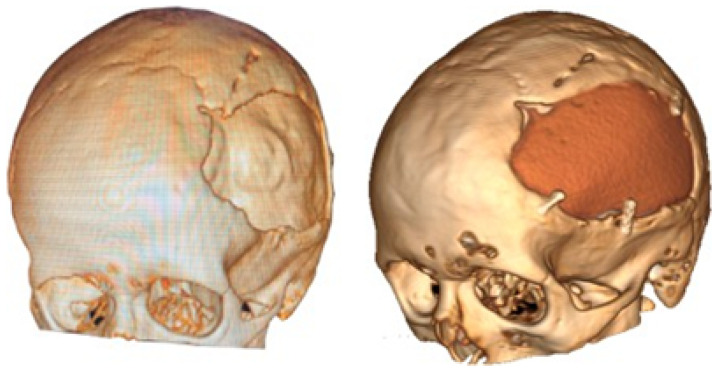
Postoperative tomographic control. The reconstruction process was successfully executed to a satisfactory degree.

**Figure 16:**
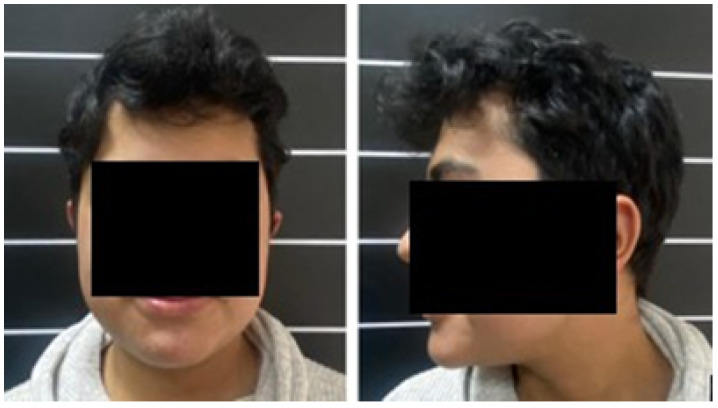
Resolution of the cranial deformity.

The postoperative results were positive and verified with the help of a new tomography, digital photography and physical examination. The complete reconstruction of the bone defect was achieved (see Figure 15 and Figure 16).

### 3.3. Case 3: Osteofibrous Dysplasia

#### 3.3.1. Diagnosis and Analysis

**Figure 17:**
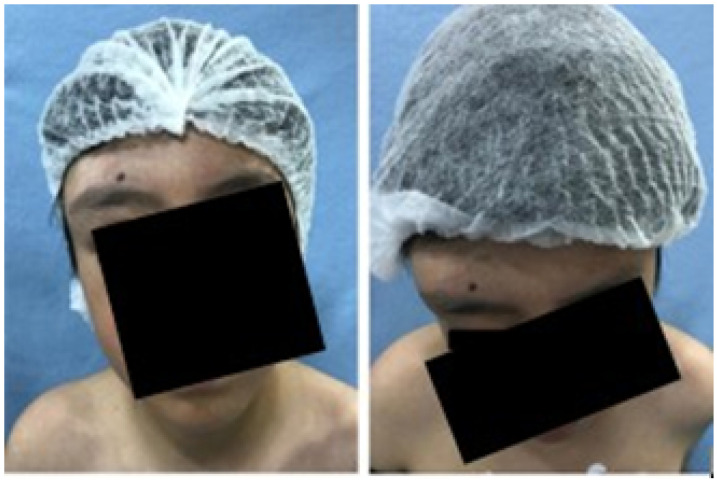
Frontal tumour, ocular proptosis, enophthalmos.

**Figure 18:**
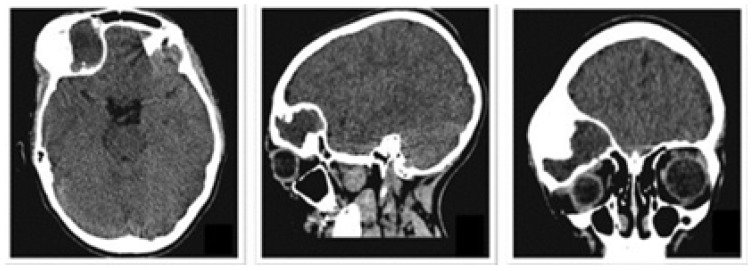
CAT: 5 cm bone lesion in the roof of the orbit, paranasal sinus and anterior vault of the skull.

**Figure 19:**
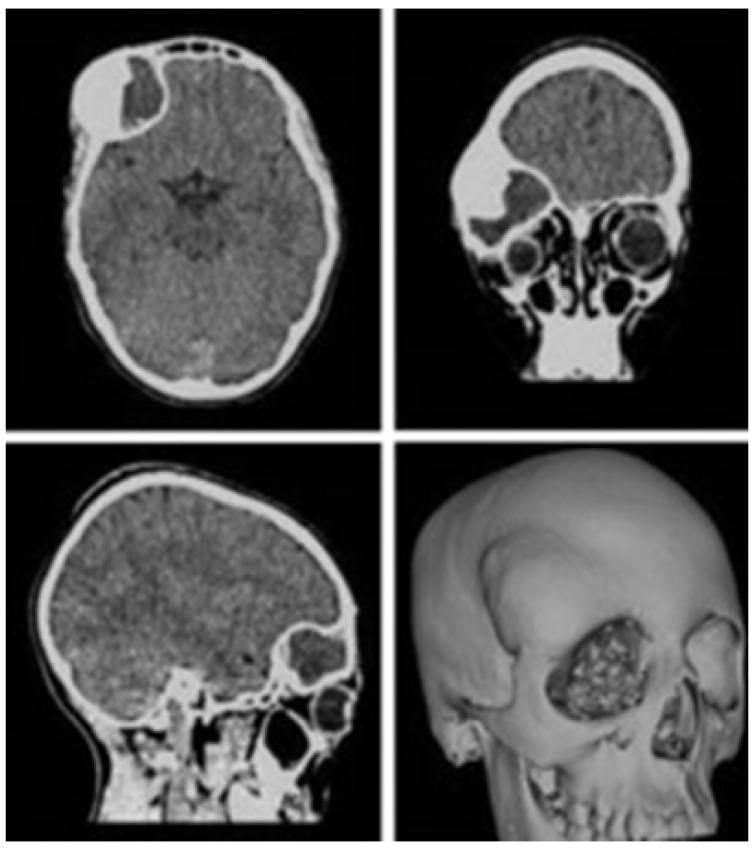
Exposure of cranial damage from the tomographic image and three-dimensional model.

The patient was an 11-year-old male who had been suffering from a hard, immobile, painless, progressively growing right frontal tumour for four years before attending the consultation. The tumour produced orbital deformity, manifesting as ocular proptosis, enophthalmos and hypotropia (see Figure 17, Figure 18 and Figure 19).

#### 3.3.2. Surgical Planning

**Figure 20:**
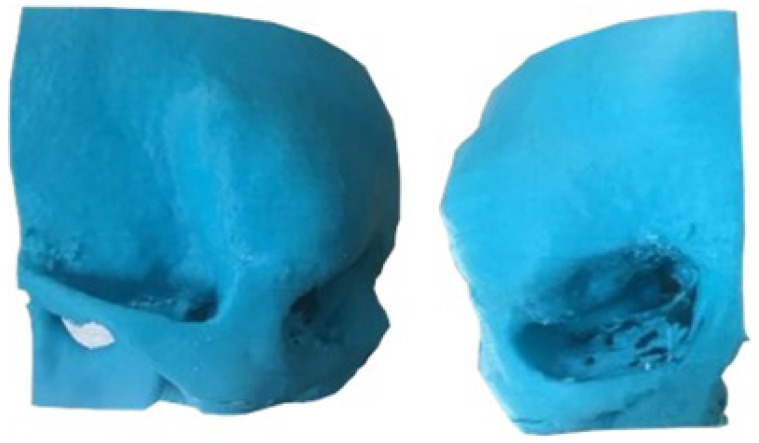
Three-dimensional printing of the anatomical model of the skull with the defect.

The defects found in the anamnesis and physical and radiological examinations were to be reconstructed using 3D digital restoration (see Figure 20). The manufacturing of anatomical test models was performed by stereolithography.

#### 3.3.3. Planning and Printing Anatomical Models

The segmentation process was facilitated by implementing a thresholding algorithm, a digital tool that enabled the isolation of the anatomical area of interest and the subsequent construction of an anatomical model for the simulated surgical procedure.

#### 3.3.4. Intraoperative Approach

**Figure 21:**
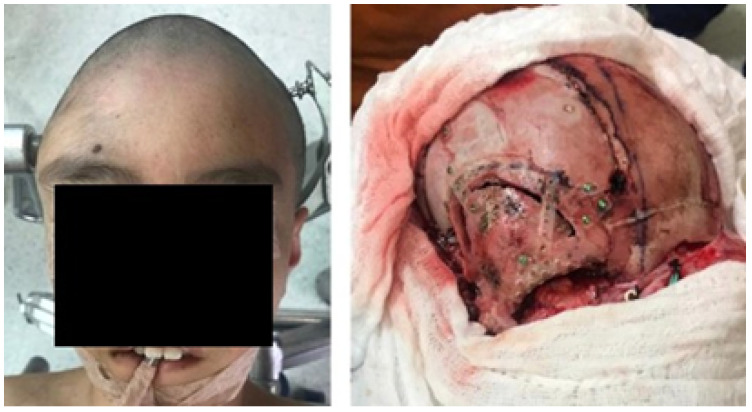
Skull fixation for actual surgery (**left**). Placement of reconstructed bone complex (**right**).

Following the selected approach from the simulated surgery, cranial osteotomies were performed in bloc, resulting in the resection of the entire affected bone complex (orbital roof, zygomatic-frontal and temporal region). The fixation procedure was performed using resorbable osteosynthesis material (see Figure 21).

#### 3.3.5. Postoperative Results

**Figure 22:**
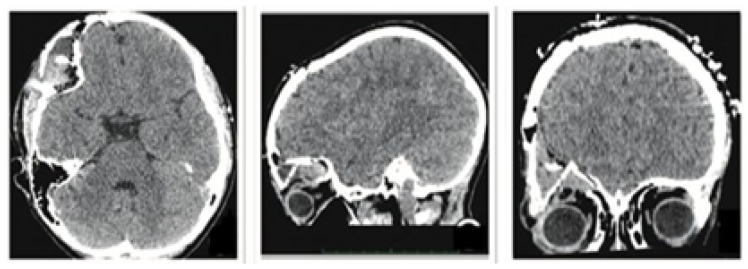
Postsurgical tomography. Removal of the cranial tumour, frontoparietal and roof orbit reconstructions are observed.

**Figure 23:**
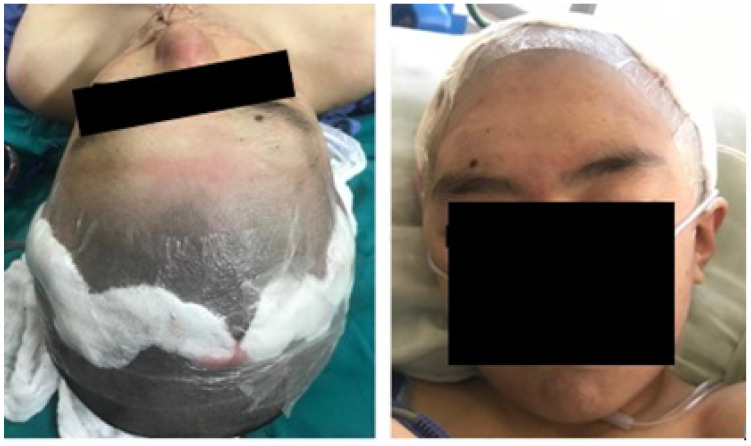
Resolution of ocular proptosis and frontal zygomatic prominence.

Postoperative results were verified with a new tomography, digital photography, and physical examination. It was possible to appreciate the complete reconstruction of the orbital roof, the anterior cranial vault and the excision of the tumour, all of which corresponded to the pre-surgical planning (see Figure 22 and Figure 23).

### 3.4. Case 4: Right Sternoclavicular Joint Tumour

#### 3.4.1. Diagnosis and Analysis

**Figure 24:**
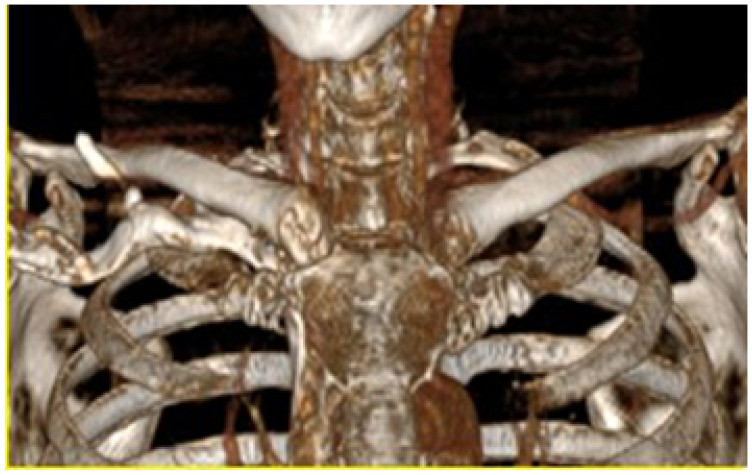
Presurgical tomography.

A 55-year-old female patient was diagnosed with osteosarcoma of the right sternoclavicular joint. This condition resulted in pain in the joint and limited the mobility of the right upper extremity (see Figure 24).

#### 3.4.2. Surgical Planning

**Figure 25:**
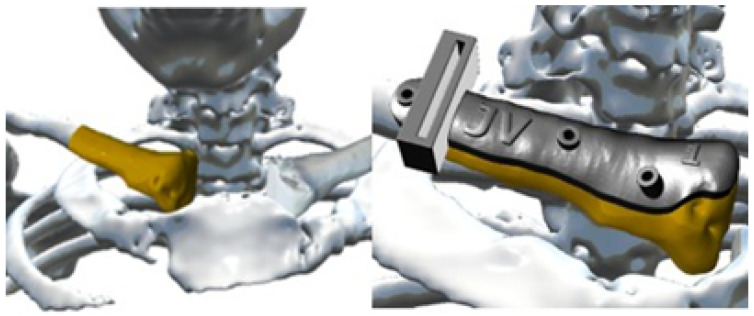
Virtual models: anatomical defect (**left**, yellow area), virtual model of the custom cutting guide (**right**, holes for fasteners and cutting slot shown).

The surgical excision of the tumour was performed through simulated surgery. The manufacturing process involved the creation of a cutting guide to facilitate the procedure and fabrication of a 3D patient-specific prosthesis. In the context of the phase of simulated surgery and the design of medical devices, a customised cutting guide was obtained from the study of tomographic images. To this end, the digital model of the anatomical surface with osteosarcoma was generated using image segmentation algorithms, the bone structure was identified, and a 5 cm notch was made in the direction of the acromial extremity to facilitate cutting (Figure 25).

**Figure 26:**
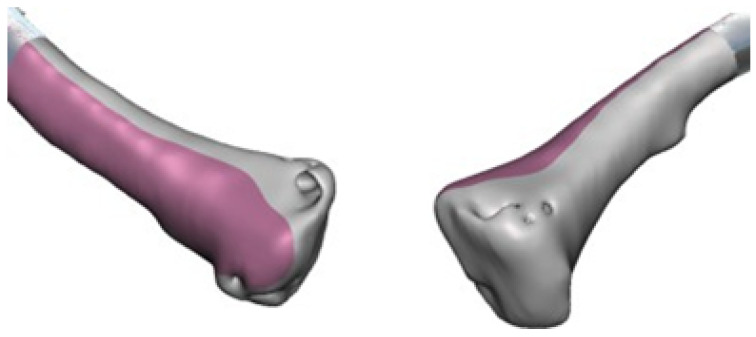
Anterior view (**left**), showing joint damage. Posterior view (**right**), defects caused by osteosarcoma.

In the case of the virtual lower clavicular replacement model, the restoration was performed on the model with osteosarcoma. Consequently, it was possible to obtain a personalised model that was both anatomically (geometrically) and structurally sound (see Figure 26).

**Figure 27:**
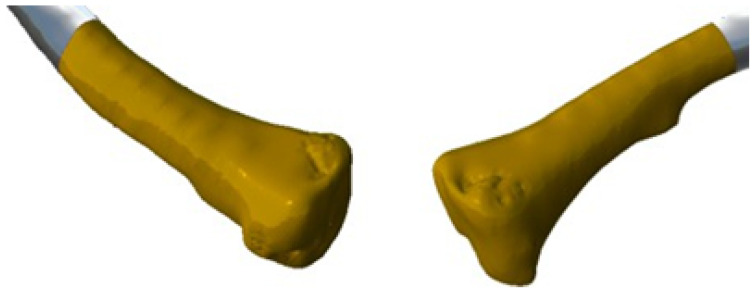
Repaired virtual model: anterior view, undamaged joint (**left**). Posterior view, without osteosarcoma (**right**).

As illustrated in Figure 27, the replacement clavicular virtual model, with the localised restorations, was employed to obtain the implant prototype through 3D additive manufacturing.

#### 3.4.3. Design and Printing of Anatomical Models

**Figure 28:**
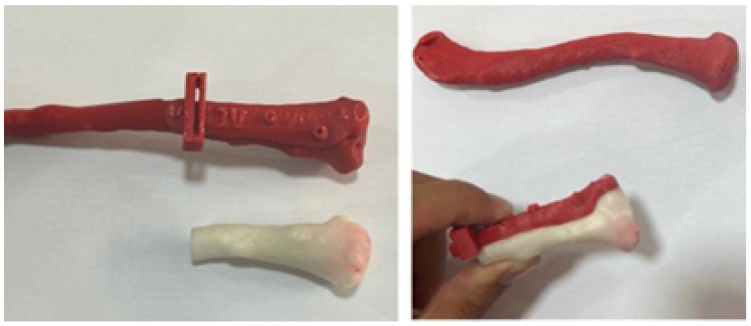
Anatomical model, cutting guide and personalised prosthesis, printed in 3D.

The 3D printed cutting guide facilitated the exeresis of the tumour with negative margins previously assessed in the tomography (Figure 28).

**Figure 29:**
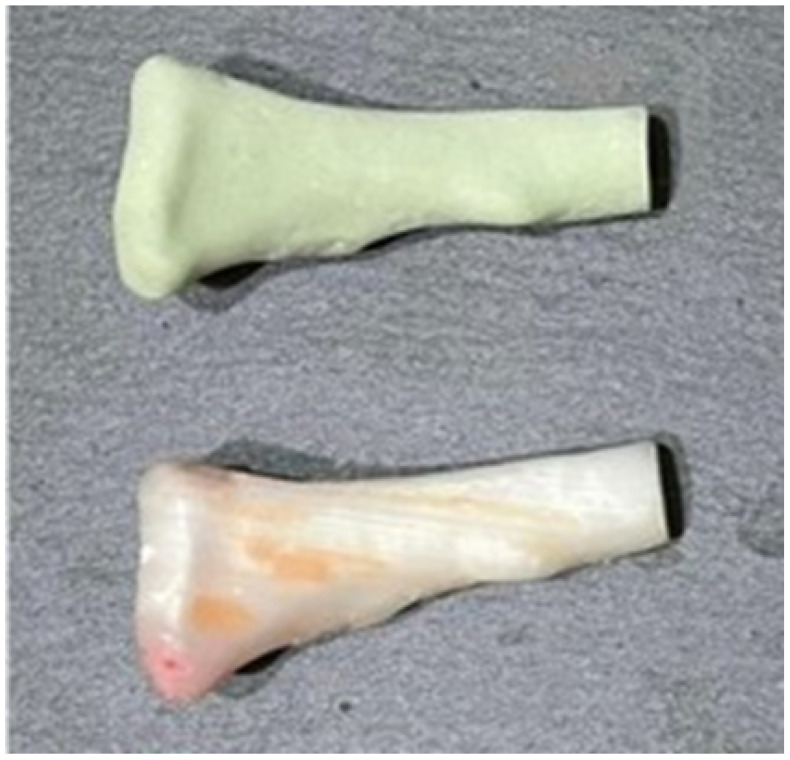
Three-dimensional printed implant anatomical model.

The three-dimensional models were used to print the test anatomical model (PLA was used as impression material), and the personalised implant for the patient was manufactured in PMMA (Figure 29).

#### 3.4.4. Intraoperative Approach

**Figure 30:**
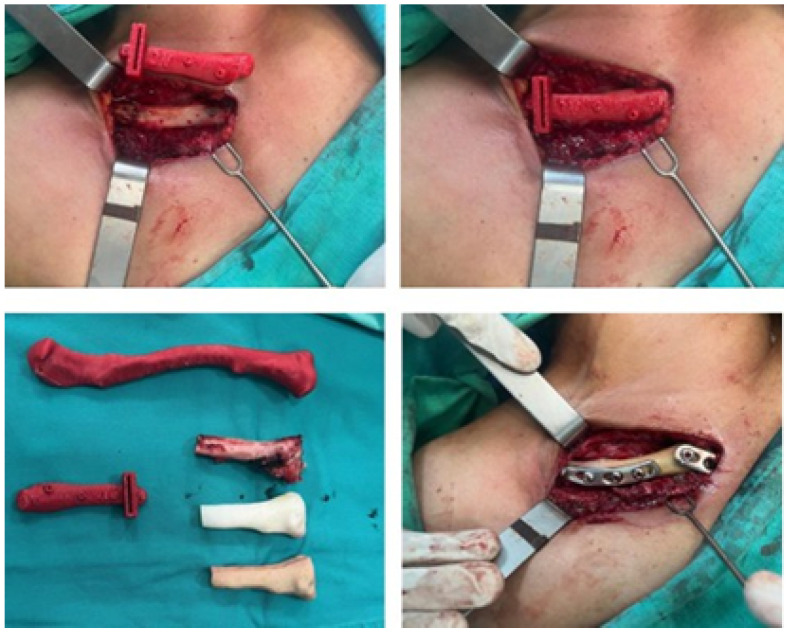
Moments of surgery: Positioning and fixing the cutting guide (**above**). The tumour was removed alongside the anatomical test model and implant (**bottom left**). Implant fixation elements (**bottom right**).

Following the surgical procedure simulation with the assistance of three-dimensional test models, actual surgery was planned and performed. This involved clavicular osteotomies, which were utilised for the excision of the tumour. In this procedure, the cutting guide was used to reconstruct the bone structure, encompassing the placement of a customised implant that was securely fixed with osteosynthetic material and covered with muscle tissue. Figure 30 illustrates various surgery stages.

#### 3.4.5. Postoperative Results

**Figure 31:**
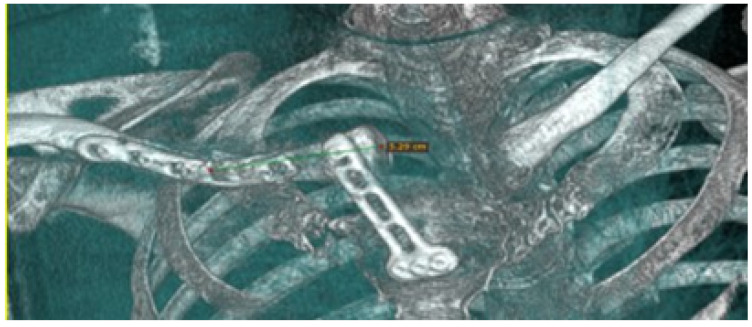
Postsurgical tomography. Reconstruction of the right sternoclavicular joint after tumour excision.

Postoperative examinations (physical and tomographic) allowed corroboration of the total reconstruction of the bone structure and the recovery of the mobility of the affected extremity (see Figure 31).

### 3.5. Case 5: Posterior Chest Tumour

#### 3.5.1. Diagnosis and Analysis

**Figure 32:**
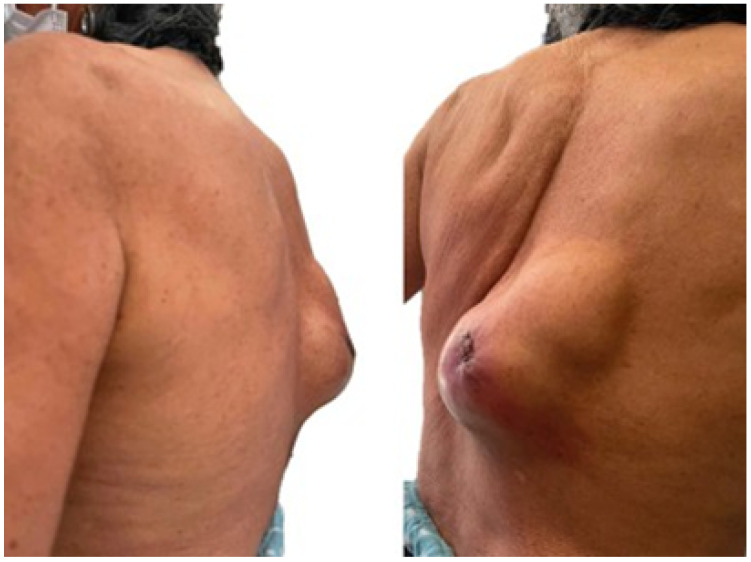
External views of the thoracic tumour.

**Figure 33:**
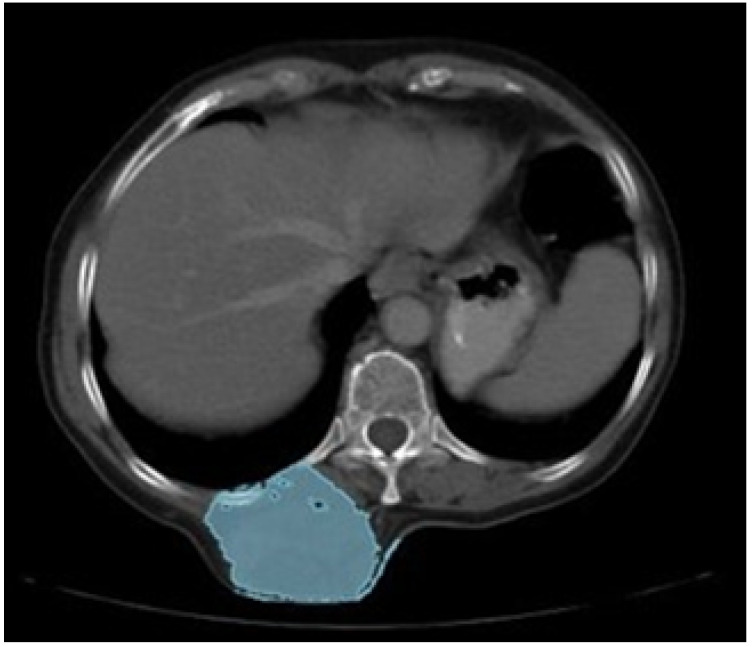
Tomographic image that reveals involvement of the skin, subcutaneous cellular tissue, soft tissues and rib grill with pleura invasion.

A 70-year-old female patient was diagnosed with a neoplasm in the right thoracic region. The lesion has been observed to affect the skin, subcutaneous cellular tissue, soft tissues and rib grill with pleura invasion (see Figure 32 and Figure 33).

#### 3.5.2. Surgical Planning

The excision of the tumour from the right posterior chest wall was conducted through simulated surgery, utilising anatomical test models that were printed in PLA (rib grill and cutting guide).

#### 3.5.3. Design and Printing of Anatomical Models

**Figure 34:**
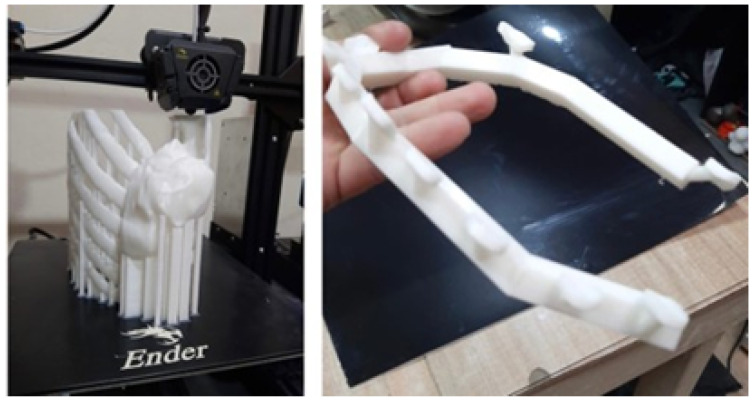
Anatomical model printing process (**left**). Cutting guide (**right**).

**Figure 35:**
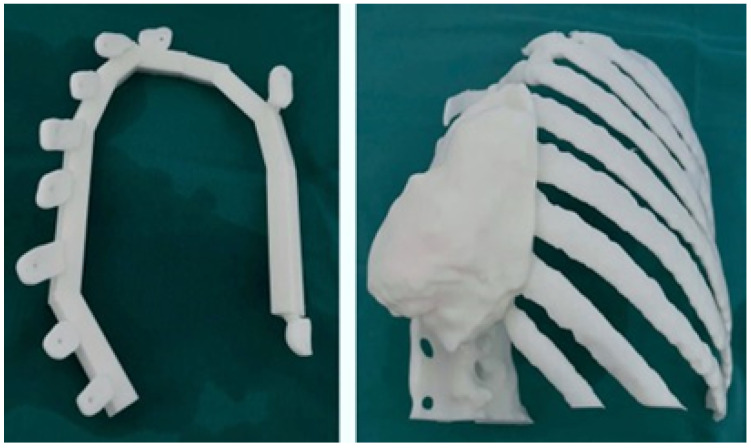
Detail of the cutting guide (**left**). Anatomical test model with the tumour (**right**).

Fabrication of the 3D anatomical test models and cutting guide was accomplished through an Ender printer. In Figure 34, the printing process and the cutting guide are shown simultaneously. In Figure 35, an additional detail of the cutting guide is demonstrated, and the anatomical test model incorporates the reproduction of the tumour.

#### 3.5.4. Intraoperative Approach

**Figure 36:**
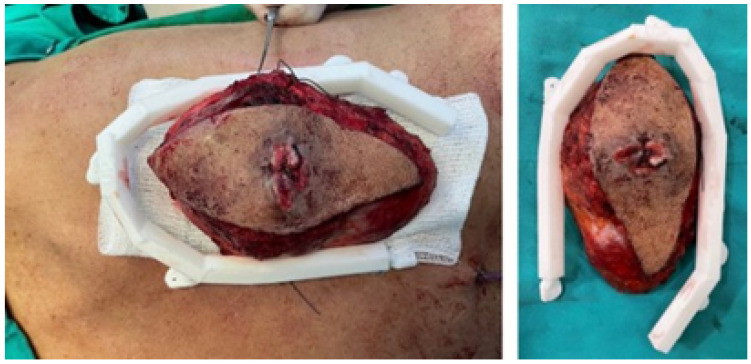
Excision of the tumour through limits marked by the cutting guide.

The use of 3D test models to simulate the surgical procedure has been demonstrated to facilitate the programming and execution of the actual intervention. In this procedure, rib osteotomies are performed for tumour excision in a bloc with the thoracic wall. The cutting guide facilitates the process. As illustrated in Figure 36, the photographic planes demonstrating the placement of the cutting guide are presented.

#### 3.5.5. Postoperative Results

**Figure 37:**
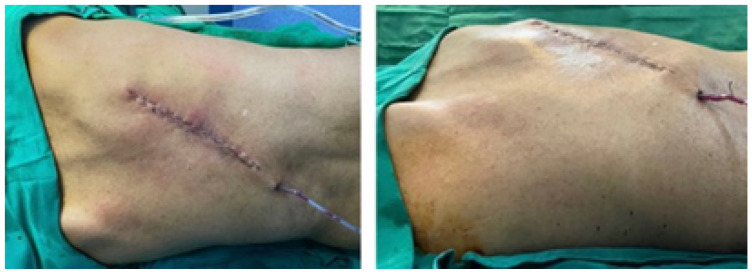
Reconstruction of the posterior chest wall.

Post-surgical examinations (tomography, digital photography and physical examination) were conducted to corroborate the complete resection of the tumour with negative margins and to verify the total reconstruction of the rib grill. Figure 37 illustrates the state of the scar some hours after surgery.

### 3.6. Case 6: Right Sternoclavicular Tumour

#### 3.6.1. Diagnosis and Analysis

**Figure 38:**
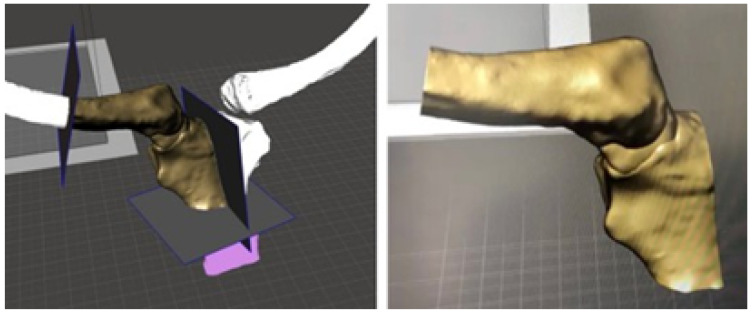
Isolation of the clavicular tumour (**left**). Clavicle and sternum reconstruction model (**right**).

A 55-year-old female patient was diagnosed with a tumour in the right sternoclavicular region, which compromised half of the sternal handle and the proximal third of the clavicle on the same side. As in the preceding cases, the three-dimensional digital model of the entire area was obtained from the tomographic images of the affected area. Figure 38 shows the process of delimiting the damaged bone structure.

#### 3.6.2. Surgical Planning

**Figure 39:**
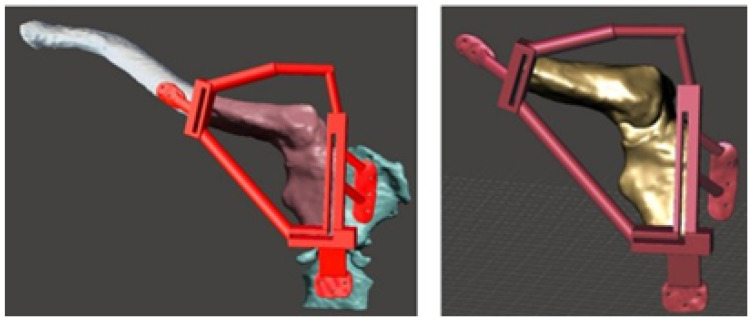
Bone segmentation model and anatomical reconstruction (**left**). Anatomical positioning of the cutting guide (**right**).

After anatomical test models and cutting guide printing, the surgical process was planned and tested. This process includes the tumour excision and bony structure reconstruction with a patient-specific 3D printed implant. As illustrated in Figure 39, the cutting guide was designed for this particular case and its placement.

#### 3.6.3. Design and Printing of Anatomical Models

**Figure 40:**
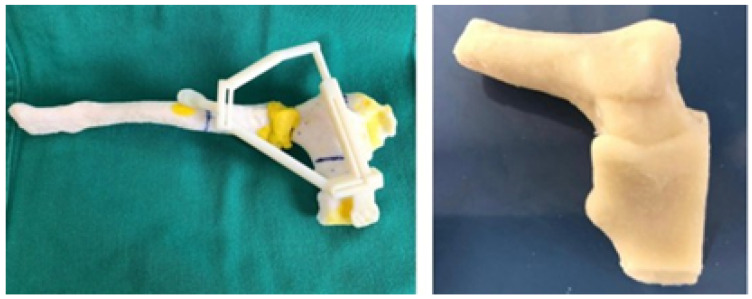
Anatomical model printed in PLA and BioMed white resin-based cutting guide (**left**). PMMA-based custom implant (**right**).

The simulation of the surgical procedure was conducted using the test anatomical model. Similarly, the test model of the cutting guide, which was printed in 3D, enabled the excision of the tumour to be simulated with negative margins that were previously assessed in the tomography. This facilitated the placement of the personalised 3D implant, which was manufactured with PMMA. As shown in Figure 40, the anatomical test model was printed with PLA, the cutting guide was manufactured with BioMed resin MED610 (this material exhibits a provisional biocompatibility that persists for 24 h), and the personalised prosthesis was printed in PMMA.

#### 3.6.4. Intraoperative Approach

**Figure 41:**
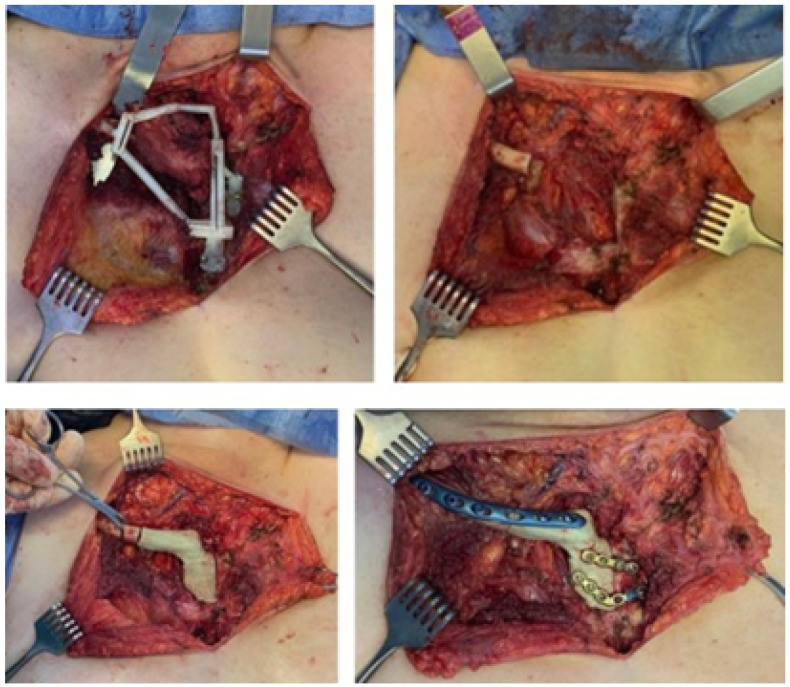
Surgery details: Positioning the cutting guide (**top**, **left**). Space left by the excision of the tumour (**top**, **right**). Placement of the custom implant (**bottom**, **left**). Implant fixation (**bottom**, **right**).

Following the simulated surgery, the respective sternoclavicular osteotomies were programmed and executed for the excision of the tumour, now with the help of the cutting guide. Following tumour resection, the reconstruction of the bone structure was initiated with the implementation of a 3D prosthesis, which was then secured using osteosynthetic material. The wound was closed and the prosthesis was covered with muscle tissue (see Figure 41).

#### 3.6.5. Postoperative Results

**Figure 42:**
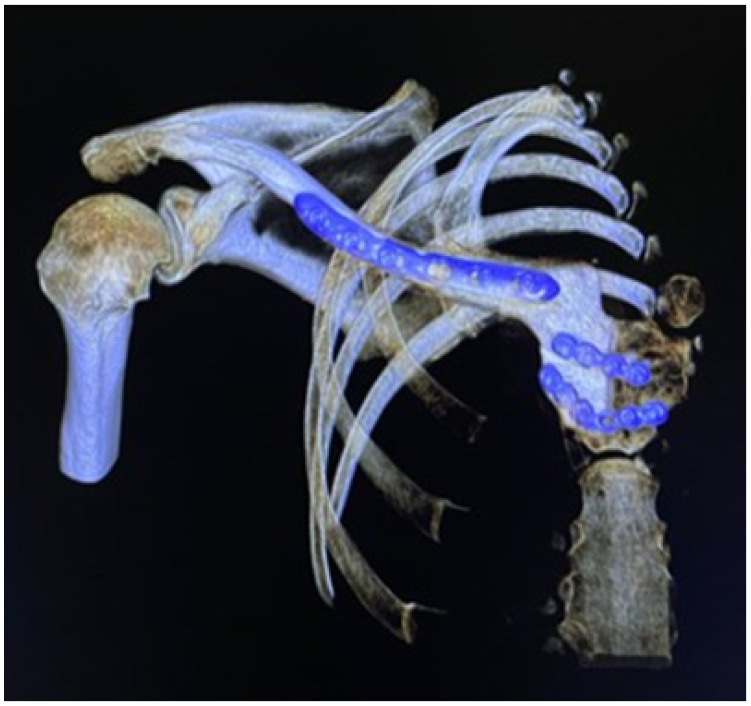
Right sternoclavicular reconstruction after tumour excision (tomographic image).

Postoperative tests (tomography, physical examination) demonstrated complete resection of the tumour, with negative margins, and reconstruction of the sternoclavicular joint with adequate functionality (see Figure 42).

### 3.7. Case 7: Metopic Craniosynostosis and Trigonocephaly

#### 3.7.1. Diagnosis and Analysis

**Figure 43:**
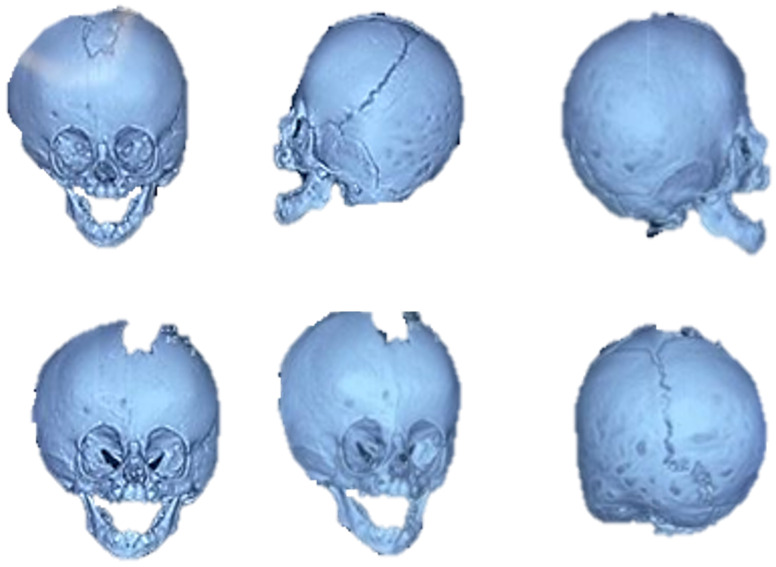
Different aspects of deformity identification (tomographic image).

A 9-month-old female patient presented with metopic craniosynostosis and trigonocephaly, characterised by premature fusion of the metopic suture, resulting in a triangular frontal deformity and possible hypertelorism. The recommended treatment in this situation is cranial remodelling surgery. Figure 43 shows different aspects of defect identification using tomographic images.

#### 3.7.2. Surgical Planning

The medical team decided to perform the surgery assisted by 3D printing technology using customised cutting guides.

#### 3.7.3. Design and Printing of Anatomical Models

**Figure 44:**
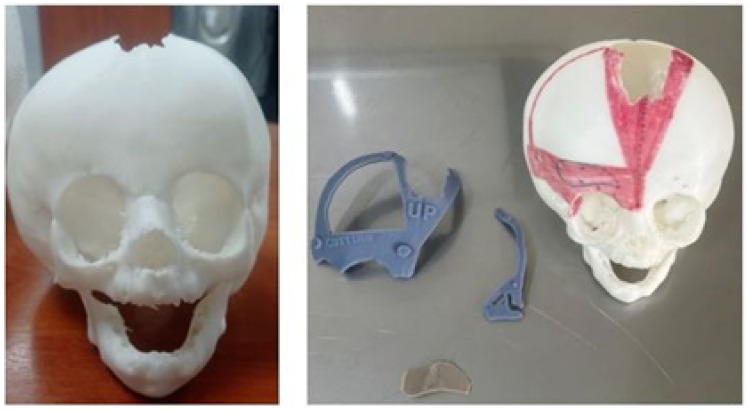
Anatomical test model of the skull (**left**). Test model of the cutting guide and planning of cuts on the cranial bone (**right**).

**Figure 45:**
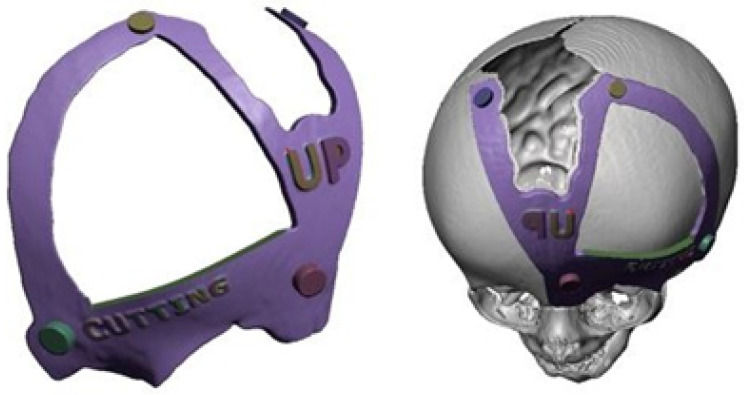
Digital models of the skull and the cutting guide.

To facilitate surgical planning and simulation, a test model of the deformed skull of the child was used in addition to the necessary cutting guide for developing the intervention. Figure 44 shows the skull’s anatomical test model and the cutting guide’s test model, both printed in PLA. And Figure 45 shows the digital models of the two medical devices.

#### 3.7.4. Intraoperative Approach

**Figure 46:**
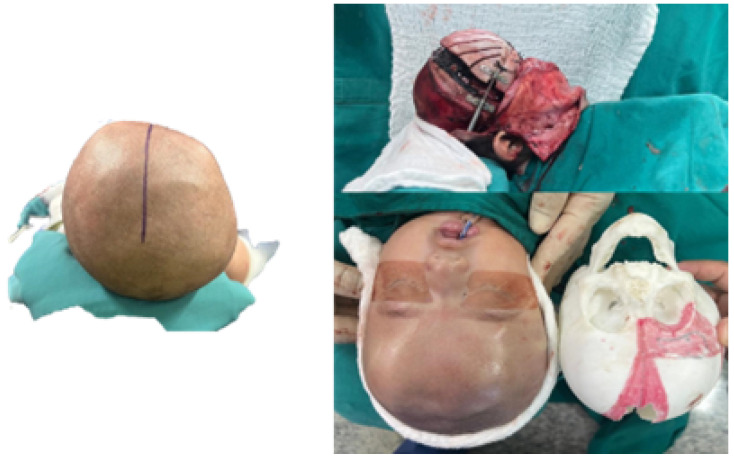
Before surgery (**left**). Bone distraction for positioning the frontal bone block (**top right**). After surgery (**bottom right**).

The patient underwent a precise, symmetrical and safe osteotomy, which reduced surgical times and improved both the aesthetic and functional results. In Figure 46, moments of the surgical process can be seen.

#### 3.7.5. Posoperative Results

With the intervention, a significant improvement in craniofacial morphology was achieved, which corresponded to the prevention of future intracranial hypertension. This procedure is intended to ensure adequate neurological development; however, it is acknowledged that some additional correction may be necessary as a result of the patient’s growth. The patient continues to receive strict medical follow-up regularly, with no abnormalities having occurred to date.

Below, in Table 3, a summary of the cases treated and the solutions applied is presented. The main clinical, surgical and follow-up variables of each case are considered, including age, diagnosis, anatomical region operated on, technique used, use of implants and surgical guides, duration of surgery, bleeding, complications, aesthetic and functional results and follow-up time.

Postoperative follow-up was developed by the medical team, according to clinical protocols, including functional, aesthetic and imaging evaluation. There are no known reports of prosthetic integration failures or the need for new intervention to date.

## 4. Results and Discussion

There is considerable information on the benefits and limitations of 3D printing for medical applications. Surveys conducted on surgeons regarding this activity have indicated that the three primary benefits of this technology are the capacity for surgical planning, enhancement in the quality of surgical outcomes and reduction in patient risks and complications. Conversely, the three principal factors that impede the utilisation of 3D printing in the public health domain are the elevated expense, the intricacy of the organisational framework required to obtain the finished product and the extended delivery time [36].

The primary advantages of implementing a planned and personalised surgical approach are safety, patient satisfaction and reduced surgical time [37,38]. In light of the great importance attributed to patient safety, the provision of a predefined cutting guide and a personalised implant has been shown to mitigate the risks associated with procedures conducted without such aids. The utilisation of the 3D printed cutting guide and implant resulted in a reduction of approximately 30% in the duration of the surgical procedure. This finding is of significant importance from a health economic perspective [39].

As demonstrated in [40,41], 3D printing technique utilisation in maxillofacial surgical interventions offers several notable clinical advantages. These include a reduced risk of injury and/or damage to the mandibular nerve, a diminished risk of mandibular fractures, a decreased risk of intra- and postoperative bleeding, and a reduced duration of surgery due to the streamlined procedure. Concerning financial implications, there are concomitant benefits. As asserted in [42], it is estimated that a substantial reduction in operating room expenses and duration can be achieved through the utilisation of a consumer-grade 3D printer. The findings of the present study indicate that a total of 58 h of annual operating room time was saved, which is equivalent to a reduction in operating room costs amounting to USD 87,000.00.

Similarly, the current state of affairs is not entirely conducive to the medical applications described. In [43,44], the authors posit that a notable disadvantage associated with 3D printed medical devices corresponds to the cutting guides’ utilisation. Specifically, they contend that erroneous placement of the bespoke guide on the bone to be manipulated can potentially result in inaccuracies. Following the results reported in this synthesis of cases, the significance of this technology for medical applications is based on the following:

1. The utilisation of scale 1:1 models for simulated surgery and trial-and-error testing with the corresponding anatomical models is pivotal in appropriate surgical approach selection and the individualised planning of surgery for each patient.
2. The additive manufacturing of surgical guides facilitates the execution of precise incisions, with sufficient margins to ensure both oncological efficacy and defect reconstruction.
3. The fabrication of custom-made prostheses for bone reconstruction and restoration circumvents the morbidity associated with the donor site and the augmented anaesthetic-surgical time, which is concomitant with freehand manufacturing. The prosthesis is custom-made to fit each patient’s specific defect, offering both anatomical and aesthetic benefits.

All factors mentioned above result in advantageous outcomes for the patient. This procedure has been shown to result in a reduction in hospital stay time, anaesthetic-surgical times, potential morbidity and mortality of the donor site and the total final cost.

Despite the favourable results obtained in this case series, it is important to recognise certain limitations inherent to the use of surgical planning and 3D printing technologies. These include possible material fatigue in long-term implants, especially in those not subjected to standardised mechanical testing processes [45].

Finally, implementation of these technologies in resource-limited settings may be hampered by economic and logistical barriers, despite demonstrated potential clinical benefit.

### 4.1. Economic Analysis of 3D Surgical Planning

Surgical planning assisted by 3D technology has demonstrated direct economic advantages, especially in complex surgeries that require anatomical precision and personalised reconstructions that would modify their initial conception towards intelligent and optimisable devices [46,47,48,49,50,51,52,53,54,55]. Its implementation allows for a significant reduction in operating time (∼30% less), minimises postoperative complications, avoids reinterventions and eliminates the need for bone grafts from donors. Although the manufacture of anatomical models and personalised cutting guides involves a moderate initial cost, this is largely offset by the savings in surgical and hospital resources, as well as the improvement in the procedure efficiency. These benefits are particularly relevant in contexts where optimisation of surgical time and patient safety are priorities. In all the cases analysed in this series, there was a shorter hospital stay, with no complications and satisfactory clinical results.

#### Implementation Costs

Regarding implementation costs, it was identified that surgical planning with 3D printing requires a moderate investment, mainly concentrated in the manufacture of anatomical test models, surgical cutting guides and personalised implants. The estimated values for each component were kept within an accessible range: between USD 15 and 30 for anatomical models, USD 50 to 80 for cutting guides, USD 100 to 200 for PMMA implants, and up to USD 850 for PEEK implants. The use of open-source software and the availability of specialised technical personnel within the medical team made it possible to optimise resources and reduce operating costs.

## 5. Conclusions

The use of computer-aided design and additive manufacturing methodologies has proven to be a highly effective approach in the design of custom implants.

Customised implants manufactured using fused deposition modelling have also been clinically proven to improve patient aesthetics and minimise surgical duration, blood loss and the risk of complications. The methodology used provides surgeons with greater discretion in selecting the best treatment option for each patient’s specific needs. It is essential to recognise the limitations of this technology and that, despite its numerous advantages, it should not replace the surgeon’s clinical judgment or technical expertise.

This innovative medical approach, in general terms, seeks to use individualised information about each patient (their genetics, lifestyle, environment, etc.) to prevent, diagnose and treat diseases more effectively.

In the future, the development of prospective studies and controlled clinical trials is recommended to systematically validate the effectiveness, safety and cost–benefit of surgical planning with 3D printing compared to conventional techniques [47]. It is also necessary to advance regulatory frameworks that facilitate the approval for using personalised medical devices and promote scalable implementation strategies in public health systems and resource-limited regions, where this technology could have a transformative impact on surgical practice.

## Figures and Tables

**Table 1:** FDM additive manufacturing characteristics and parameters.

Characteristics and Manufacturing Parameters	Fused Deposition Modelling (FDM)
Company and model	Creality CR-X Pro (2019 Updated)
Maximum build envelope	300 × 300 × 400 mm^3^
Nozzle diameter	0.4mm
Positioning resolution (X−Y−Z)	1.25 μm/1.25 μm/1 μm
Selected layer thickness	0.1 mm
Printed filament line width	0.4 mm

**Table 2:** Characteristics of the polymers utilised in this study.

Characteristics	PLA (FDM)	PMMA	Resin PolyJet	PEEK
Polymer	Thermoplastic	Thermoplastic	Photopolymer	Thermoplastic
Manufacturer	Creality HP	Veracril, New Stetic S.A.	Stratasys	Evonik Corporation
Commercial	HP-PLA	PMMA	MED610	Vestakeep PEEK
Color	White (Bone)	Transparent	Transparent	Light gray
Density	1.23 kg m^−3^	1.15–1.19 kg m^−3^	1.17–1.18 kg m^−3^	1300 kg m^−3^
Tensile strength	52 MPa	30–50 MPa	50–65 MPa	90–100 MPa
Print/molding temperature	190–220 °C (±5 °C)	60–80 °C	45–50 °C	260–300 °C
Filament diameter	1.75 mm	-	-	1.75 mm
Printed diameter	0.55 mm	-	-	0.55 mm

**Table 3:** Summary of patients treated and solutions provided.

Variable/Case	Case 1	Case 2	Case 3	Case 4	Case 5	Case 6	Case 7
**Age **	2 years old	10 years old	11 years old	55 years old	70 years old	55 years old	9 months old
**Sex**	Female	Male	Male	Female	Female	Female	Female
**Diagnosis**	Sequela of craniocerebral trauma	Post-trauma cranial collapse	Osteofibrous dysplasia	Sternoclavicular osteosarcoma	Posterior thoracic tumour	Sternoclavicular tumour	Metopic craniosynostosis and trigonocephaly
**Anatomical region**	Left temporoparietal and orbital	Left temporoparietal-occipital	Right frontal and orbital roof	Right sternoclavicular joint	Right posterior chest wall	Right sternum and clavicle	Frontal bone and metopic region
**Type of intervention**	Osteotomy, resection, reconstruction	Cranioplasty	Tumour resection, craniofacial reconstruction	En bloc resection, prosthesis	En bloc rib resection, thoracic reconstruction	Resection, sternoclavicular reconstruction	Cranial remodelling assisted with surgical guide
**Medical device**	Orbital prosthesis	Custom prosthesis	Reconstructed bone complex	Sternoclavicular implant	Custom rib implant	Sternoclavicular implant	Custom cutting guide
**Implant material**	Own bone	PMMA	Medical PEEK	PMMA	PMMA	PMMA	Not applicable
**Surgical guide (yes/no)**	Yes (3D model for planning)	Yes (3D model for planning)	Yes (3D model for planning)	Yes (3D model for planning)	Yes (3D model for planning)	Yes (3D model for planning)	Yes (3D model for planning and surgery)
**Approximate surgery time**	2.2 h	1.15 h	1.45 h	2.3 h	1.55 h	2.3 h	1.5 h
**Aesthetic result**	Improvement of proptosis and cranial symmetry	Cranial contour restoration	Resolution of frontal and orbital deformity	Adequate anatomical reconstruction	Thoracic reconstruction	Restored sternoclavicular symmetry	Corrección morfológica craneofacial
**Postoperative functionality**	Adequate	Adequate	Adequate	Improved mobility of the right arm	Thoracic and respiratory stability	Preserved joint functionality	Prevention of intracranial hypertension
**Medical follow-up time**	Not specified (immediate control by TAC)	Not specified (immediate control by TAC)	Not specified (immediate control by TAC)	Not specified	Not specified	Not specified	Regular pediatric follow-up (ongoing)
**Additional comments**	Good clinical and aesthetic result	Good clinical and aesthetic result	Complete reconstruction of orbit and skull	Use of personalised cutting guide for resection	Resection with negative margins	Use of biocompatible resin in surgical guide	Effective technique in childhood with good prognosis

## Data Availability

The original contributions presented in this study are included in the article. Further inquiries can be directed to the corresponding author.
